# Stimulants and Sedatives

**Published:** 1898-03

**Authors:** 


ARTICLE XI.

### STIMULANTS AND SEDATIVES.

To use drugs intelligently, the physician needs to be a close observer and a good reasoner. In the human system exactly opposite causes sometimes produce very similar symptoms, and a medicine which will remedy one cause excites the other. In such cases careful inquiry into the patient's daily life, and a study of his temperament and idiosyncrasies, will save much experimenting on the part of the Doctor and impatience and disgust in the case of the patient.

For example, the nervous system may suffer from over or insufficient use. Where the trouble is abuse, the nervous system is hyperemic, very irritable, its nutrition is unstable, there is excessive waste on slight effort, which necessitates constant efforts at repair. In such a case, to give nerve stimulants such as strychnia, is suicidal. What is needed is a combination of nerve sedative and circulatory stimulant. The first lessons nervous excitability, the second brings fresh blood to repair waste and carry off broken down tissue elements. A combination which works exceedingly well is equal parts of Peacock's Bromides and Celerina, in drachm doses several times a day. Another good mixture for temporary use is Papine and aromatic spirits of ammonia, ten drops of the former to thirty of the latter.

In cases where the nervous system requires stimulation, we find torpidity, lethargy. The individual, while apparently in good condition, eating and sleeping well, looks, moves and feels as though he had been drugged. Has no ambition; content to sit around and vegetate. In these cases Celerina alone, in teaspoonful doses every three or four hours, will brighten up these patients wonderfully.

A physician remarked to the Editor, recently, that Fellows' Syr. Hypophos. Comp. was a very good remedy, but he could not always tell whether it was indicated in a given case or not. That sometimes it did great good, but occasionally seemed to make the patient worse. This is because it is a tonic to the nervous system, and where the latter has already been stimulated by over-work to the point of exhaustion, sedation and recuperation must precede the use of tonics.

What is true of the nervous system applies equally to the digestive system, the genita-urinary system, etc. It would be a great mistake to treat a stomach in open, angry revolt, rejecting everything put into it, and pouring forth quantities of perverted secretions as we would a lax, sluggish, atonic organ, coated with mucus. So, too, it would be foolish to give a renal stimulant like buchu to a kidney badly congested, secreting blood and pus, but the indolent, catarrhal kidney might require stimulation. And right here we desire to remark the unwisdom of giving alkalies to persons suffering from Bright's disease, diabetes, malaria, uric acid diathesis, or any other condition in which the skin is inactive. They increase the renal congestion, and make the patient worse.

To recognize widely varying pathology, often masked by similar symptoms, needs much insight and thought on the part of the Doctor. He must study indications carefully, see that his drugs are pure, and that no substitution is practiced upon him, in order to avoid all possible sources of error.—*Medical Brief.*

